# Design of a Protocol for Soil-Transmitted Helminths (in Light of the Nematode *Toxocara canis*) DNA Extraction from Feces by Combining Commercially Available Solutions

**DOI:** 10.3390/diagnostics13132156

**Published:** 2023-06-24

**Authors:** Alexander A. Devyatov, Ekaterina E. Davydova, Andrey R. Luparev, Sofia A. Karseka, Anna K. Shuryaeva, Angelica V. Zagainova, German A. Shipulin

**Affiliations:** Federal State Budgetary Institution “Centre for Strategic Planning and Management of Biomedical Health Risks” of the Federal Medical Biological Agency, 10 bld 1, Pogodinskaya Str., 119121 Moscow, Russia; edavydova@cspmz.ru (E.E.D.); aluparev@cspmz.ru (A.R.L.); skarseka@cspmz.ru (S.A.K.); ashuryaeva@cspmz.ru (A.K.S.); azagaynova@cspmz.ru (A.V.Z.); shipulin@cspfmba.ru (G.A.S.)

**Keywords:** helminths, DNA extraction protocol, feces, commercially available solutions

## Abstract

One of the main challenges for the mass introduction of the molecular diagnostics of soil-transmitted helminths (STHs) into clinical practice is the lack of a generally recognized effective method for isolating parasitic DNA from fecal samples. In the present study, we assessed the effects of various pretreatment procedures on the efficiency of removing PCR inhibitors and extracting *Toxocara canis* DNA from feces. We evaluated the effectiveness of four destructive methods (bead beating, the action of temperature-dependent enzymes, freeze-heat cycles, and incubation in a lysis buffer) on the integrity of *T. canis* eggs and the efficiency of DNA extraction. Also, we evaluated the effects of prewashes and the use of commercial concentrators on DNA extraction from fecal samples contaminated with *T. canis* eggs. A bead beating procedure was sufficient to destroy the *T. canis* eggs, while the effects of enzymes and freeze-heat cycles did not lead to a significant destruction of the eggs or the release of *Toxocara* DNA. Helminth DNA isolation protocols that do not include a bead beating step are not preferred. The preconcentration of STH eggs from feces using a commercial concentrator and subsequent washing can significantly increase the yield of DNA from STHs and reduce PCR inhibition.

## 1. Introduction

The are currently approximately one billion people worldwide that are infected with soil-transmitted helminths (STHs) [1]. STH infection can lead to health problems such as chronic blood loss leading to anemia, the malabsorption of nutrients, loss of appetite, lethargy, growth retardation in children, and cognitive decline [2,3,4]. Currently, the main methods for detecting STHs are microscopic methods [5,6,7]. With advantages such as a relatively low cost and no need for expensive and difficult-to-maintain equipment, these methods are also quite subjective and require a high qualification of the performer, which leads to frequent false negative results [8,9,10,11]. Thus, there is a need to design more sensitive, less time-consuming, and high-throughput methods for detecting STHs.

Recently, an increasing number of works have appeared on the use of molecular methods for the diagnosis of STHs. In most studies which have compared the sensitivities of methods using microscopy and molecular diagnostic methods, the latter had higher sensitivity [12]. In addition, molecular methods are very specific compared to microscopy and make it possible to distinguish between morphologically related species [9,13]. Molecular methods also make it possible to detect changes in the genome which are associated with resistance to anthelmintics [14].

Having said that, the design of highly sensitive methods for the molecular diagnoss of helminthiases requires solving some complex technical problems. First, the shell of helminth eggs is quite strong, and it is necessary to select sufficiently harsh conditions to disrupt it while not damaging the helminth DNA [12]. The second problem is the complex composition of the tested clinical material. Feces is a multi-component matrix containing a variety of compounds, including bile acids and other substances that inhibit amplification reactions [15]. To solve these problems, various research groups have proposed either proprietary protocols [16,17] or used commercially available nucleic acid isolation kits modified with additional processing steps for clinical material, such as adding sorbents, freeze-thaw cycles, heating, mechanical processing with beads, treatment with enzymes, etc. [18,19,20]. Nevertheless, the effectiveness of most of the additional processing steps that are used used is not completely clear, and the lack of generally recognized methods for isolating the DNA of STHs leads to a large scatter in the assessment of the sensitivity of molecular methods for diagnosing diseases caused by these parasites [12].

*Toxocara canis* (Werner, 1782) (*Ascaridida*, *Toxocaridae*) is a neglected zoonotic parasite which threatens the health of dogs and humans worldwide [21]. Despite the fact that *T. canis* does not produce eggs in the human intestine, its’ eggs can be considered as a model object, because they have a similar composition and structure to STHs found in human feces [22,23].

Therefore, the present study aimed to assess the effectiveness of the most commonly used methods for the destruction and concentration of STHs eggs on the efficiency of DNA extraction. *T. canis* eggs were used as a model object. Polymerase chain reaction and microscopy were used to evaluate the DNA yield and the number of undamaged eggs, respectively. As a result of this study, we present an efficient protocol for STHs’ DNA extraction using commercially available solutions.

## 2. Materials and Methods

### 2.1. Ethics Statement

Fecal samples were obtained from the G.N. Speransky Children’s City Clinical Hospital No 9, Moscow, Russia. Ethical approval for using clinical samples for research purposes was obtained from the local clinical hospital ethics committee (protocol no. 44, 19 April 2022).

### 2.2. Preparation of T. canis Egg Suspension

A suspension of *Toxocara* eggs in saline was provided by the Laboratory of Microbiology and Parasitology of the Federal State Budgetary Institution “Centre for Strategic Planning and Management of Biomedical Health Risks” of the Federal Medical Biological Agency (Moscow, Russia). The *Toxocara* eggs were concentrated from the feces of infected dogs and then resuspended in saline (0.9% NaCl). The concentration of the eggs in the suspension was assessed visually using a microscope.

The concentration of the *Toxocara* eggs was adjusted to 1500 eggs/mL. If the concentration of eggs was greater than this value, the suspension was diluted to the desired concentration with saline. If the concentration of eggs in the suspension was less than 1500 eggs/mL, the suspension was spun at 10,000× *g* for 5 min to increase the concentration, after which the required amount of supernatant was taken out, and then the sediment containing the eggs was resuspended.

#### Measurement of *Toxocara canis* Egg Concentration by Microscopy

The concentration of eggs in the suspension was assessed visually using a Micromed 1 microscope (Micromed, Saint Petersburg, Russia). Ten microliters of the suspension were applied to a glass microscope slide, after which it was covered with a cover slip. The entire preparation was viewed under a microscope using 40× magnification, and the number of eggs in the preparation was counted. This procedure was repeated 10 times, after which the arithmetic average of the number of eggs for 10 specimens was calculated, and the result was multiplied by 100 to obtain the concentration of eggs per milliliter.

### 2.3. Obtaining Model Samples of Feces Contaminated with T. canis Eggs

Fecal samples (*n* = 52) from patients without signs of helminth infection were obtained from the G.N. Speransky Children’s City Clinical Hospital No. 9 (Moscow, Russia). The amount of feces in each sample was at least 7 g.

Fecal samples contaminated with *T. canis* eggs at concentrations of 1000, 500, 50, 10, and 5 eggs per gram of feces were prepared by adding the appropriate amount of *Toxocara* egg suspension to the sample. In experiments on the effects of preliminary washings and the concentration of helminth eggs on the results of PCR after the isolation of *T. canis* DNA from native feces, fecal samples with a concentration of 1000 eggs/g were used. In the experiment to evaluate the minimum detectable number of *T. canis* eggs in native feces, fecal samples with concentrations of 1000, 500, 50, 10, and 5 eggs/g were used.

### 2.4. Comparison of the Effect of Various Destructive Methods on the Integrity of T. canis Eggs and the Efficiency of DNA Extraction

To assess the effects of various methods on the integrity of eggs and the efficiency of *T. canis* DNA extraction, we selected four destructive methods: freeze-heat cycles, bead beating, the CD1 lysing buffer of the QIAamp PowerFecal Pro kit (QIAGEN GmbH, Hilden, Germany), and temperature-dependent enzymes from the forensicGEM Sperm kit (MicroGEM International PLC, Southampton, United Kingdom). 

Egg integrity was assessed using a light microscope by counting the number of remaining undamaged eggs in the suspension. For the experiment, five 50 µL aliquots of *Toxocara* egg suspension (1500 eggs/mL) were used, which were diluted with saline to a final volume of 100 µL. Tubes with aliquots of *Toxocara* egg suspension were labeled according to the exposure method: Susp—pure suspension (control); CD1—incubation in CD1 buffer of the QIAamp PowerFecal Pro kit; Frz-ht—four cycles of freezing-heating; Enzs—temperature-dependent enzymes from the forensicGEM Sperm kit; BB—bead beating with QIAamp PowerFecal Pro ceramic beads.

Evaluation of the destruction methods of *Toxocara* eggs for their ability to increase the yield of DNA was carried out using PCR. The volume of DNA elution for all samples was 100 µL.

The sample preparation protocols are given in Table 1.

As seen from the above protocols for preparing samples for microscopic examination, the final volume of the resuspended sediments of the homogenates after all manipulations was the same for all aliquots at 100 µL. Since the CD1, Enzs, and BB samples had to be centrifuged to remove the excess fluid volume, the Susp and Frz-ht samples were subjected to the same centrifugation procedure to take into account the possible effect of centrifugation on the integrity of the helminth eggs. The number of undamaged eggs in the resulting sediments of the homogenates was assessed under a microscope, as described above. For each sample, 8 specimens were made, after which the counting data for each specimen were analyzed.

#### qPCR

Primers for the *T. canis* internal transcribed spacer 1 (ITS1) conserved region were designed using the NCBI Primer Blast web service [25] and based on the ITS1 nucleotide sequences of the *Toxocara* species available in the NCBI Nucleotide Database [26]. The resulting primer pairs, together with the nucleotide sequences of *Toxocara* and related roundworm species, were aligned using the Unipro Ugene software, version 41.0, using the Clustal W algorithm. Based on the obtained alignments, a TaqMan probe was selected following the standard requirements for the selection of oligonucleotide primers and TaqMan probes [27,28]. The thermodynamic characteristics of the primers, the fluorescent probe, and their secondary structures were evaluated using the Themfold Web Server online service [29]. Synthesis of the primers and probes was carried out by JSC Genterra.

The primers and probe sequences are shown in Table 2.

The reaction mixture with a total volume of 25 µL contained the following components: 10 µL of extracted DNA solution, 3.2 nM of each primer, 2.0 nM probe, and 5 µL of 5× Genta qPCR MasterMix (JSC Genterra, Moscow, Russia). PCR was performed on a CFX96 RealTime PCR amplification platform (Bio-Rad, Hercules, CA, USA).

The amplification program included the following stages of thermal cycling: 95 °C for 15 min, followed by 45 cycles of 95 °C for 15 s, 58 °C for 30 s, and 72 °C for 15 s. The fluorescence accumulation signal of the target DNA was recorded using the channel for the FAM fluorophore.

The result was evaluated by the threshold method, determining the threshold cycles of Ct amplification by the intersection of the fluorescence curve with the threshold line set in the middle of the exponential section of the fluorescence increase graph on a logarithmic scale. The threshold line was set at a level corresponding to 10–20% of the average maximum fluorescence level obtained for the positive samples in the last amplification cycle.

### 2.5. Study of the Influence of Preliminary Washings and Concentration of Helminth Eggs on the Results of PCR

#### 2.5.1. DNA Extraction

For the experiment on the effect of preliminary washings on the PCR results from the extraction of *T. canis* DNA from native feces, aliquots of fecal samples contaminated with *Toxocara* eggs at a concentration of 1000 eggs/g were preliminarily washed twice with 0.1% Tween-20 (Merck Life Science, Darmstadt, Germany) diluted in phosphate-buffered saline (VWR international LLC, Solon, OH, USA). These samples constituted experimental Group W. The reference groups were fecal samples contaminated with *Toxocara* eggs without prewash (Fec), as well as samples of a pure suspension of *Toxocara* eggs (Pure). The DNA extraction protocols for the experimental groups are shown in Table 3. The DNA elution volume for all samples was 100 µL.

To assess the effect of using parasite concentrators on the PCR results, aliquots of fecal samples contaminated with *Toxocara* eggs at a concentration of 1000 eggs/g were preconcentrated using Apacor Mini Parasep concentrators (Apacor, Wokingham, UK), after which they were concentrated once, then washed with a 0.1% solution of Tween-20 in phosphate-buffered saline to wash any formalin out of the obtained concentrates. These samples constituted the W + PS experimental group. The reference groups were samples of feces contaminated with *Toxocara* eggs, previously washed twice with Tween-20 (W) solution, as well as samples of a pure suspension of *Toxocara* eggs (Pure). The protocols for DNA extraction for the Pure and W experimental groups correspond to the protocols given in Table 3. For the W + PS group, the following protocol was used:

1. Approximately 500 mg of feces contaminated with *Toxocara* eggs at a concentration of 1000 eggs/g was added to the sample collection unit of the Apacor Mini Parasep concentrators.
2. The samples were mixed and centrifuged following the manufacturer’s instructions for the concentrators (EU Protocol v3.0 September 2017) (“Apacor Mini Parasep SF EU Protocol”, n.d.).
3. A total of 1.5 mL of a 0.1% solution of Tween-20 in PBS was added to the sediments, after which the tubes were vortexed until the feces were completely dissolved.
4. The tubes were centrifuged for 5 min at 5000× *g*, after which the supernatant was merged.
5. The sediment was resuspended in 800 µL of CD1 buffer from the QIAamp PowerFecal Pro kit and thenvortexed, after which the contents of the concentrator were transferred to clean PowerBead Pro Tubes from the QIAamp PowerFecal Pro kit.
6. Further stages of DNA extraction were carried out according to the manufacturer’s instructions for the QIAamp PowerFecal Pro kit (“QIAamp PowerFecal Pro DNA Kit Handbook”, n.d.), starting from step 3 (the stage of sample centrifugation after homogenization).

The volume of DNA elution for all samples was 100 µL.

#### 2.5.2. qPCR Results Data Processing

Real-time PCR was performed as described above. In these experiments, together with the threshold amplification cycles Ct, the maximum fluorescence intensity (ΔRFU) was determined, which was calculated for each sample as the difference between the fluorescence intensity at the end of the PCR (on the 45th amplification cycle) and at the beginning (on the 10th amplification cycle), when the signal fluorescence had stabilized and the exponential growth phase of fluorescence had not yet begun.

### 2.6. Statistical Data Analysis

Statistical data analysis was performed using the Statistica (version 12.6) software package (TIBCO Software Inc., Palo Alto, CA, USA). We used the functions of the “Nonparametric statistics” section. The “Mann–Whitney U Test” function was used to evaluate the differences between the two groups. To assess the differences between three or more groups, the functions “Summary: Kruskal–Wallis ANOVA & median test” and “Multiple comparisons of mean ranks for all groups” were used, where the dependent variables were Ct or ΔRFU values, and the experimental groups were independent variables. Comparisons of the PCR parameters were carried out within one setting. Differences with a significance level of *p* ≤ 0.05 were recognized as statistically significant.

## 3. Results

### 3.1. Comparison of Methods for Destroying T. canis Eggs Using Microscopy

**Figure 1:**
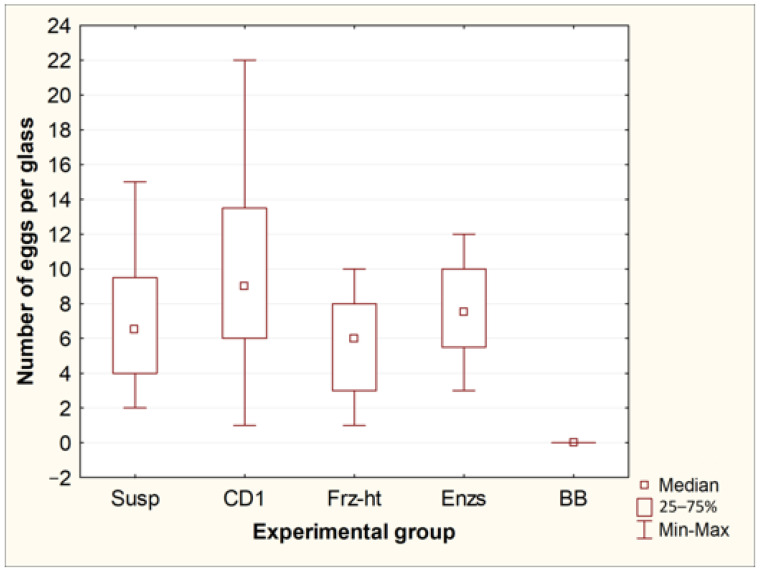
Comparison of the number of whole eggs of *T. canis* after processing by different methods. Susp—initial suspension of eggs (control, *n* = 8); CD1—incubation in CD1 buffer from the QIAamp PowerFecal Pro kit (*n* = 8); Frz-ht—freeze-heat cycles (*n* = 8); Enzs—incubation with temperature-dependent enzymes from the forensicGEM Sperm kit (*n* = 8); BB—bead beating with beads from the QIAamp PowerFecal Pro kit (*n* = 8).

**Figure 2:**
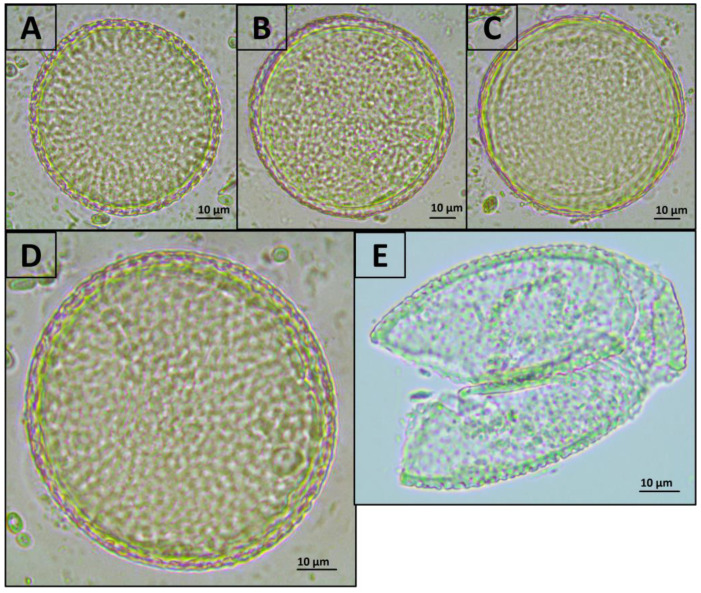
Representative photos of *T. canis* eggs after treatment with various methods (400× magnification). (**A**) Initial suspension of eggs; (**B**) incubation in CD1 buffer from the QIAamp PowerFecal Pro kit; (**C**) freeze-heat cycles; (**D**) treatment with temperature-dependent enzymes from the forensicGEM Sperm kit; (**E**) bead beating with beads from the QIAamp PowerFecal Pro kit. The colour that differs in part E in the figure is onlythe result of the open aperture of the microscope.

Exposure of *Toxocara* egg suspension to freeze-heat cycles, temperature-dependent enzymes from the forensicGEM Sperm kit, and incubation with CD1 lysis buffer from the QIAamp PowerFecal Pro kit did not lead to significant changes in the number of undamaged eggs in the suspension. However, after bead beating with beads from the QIAamp PowerFecal Pro kit, no undamaged eggs were found in the samples, while several objects were found that were presumably broken eggs (Figure 1 and Figure 2).

### 3.2. Comparison of Various Destructive Methods on the Yield of DNA from T. canis Eggs by PCR 

**Figure 3:**
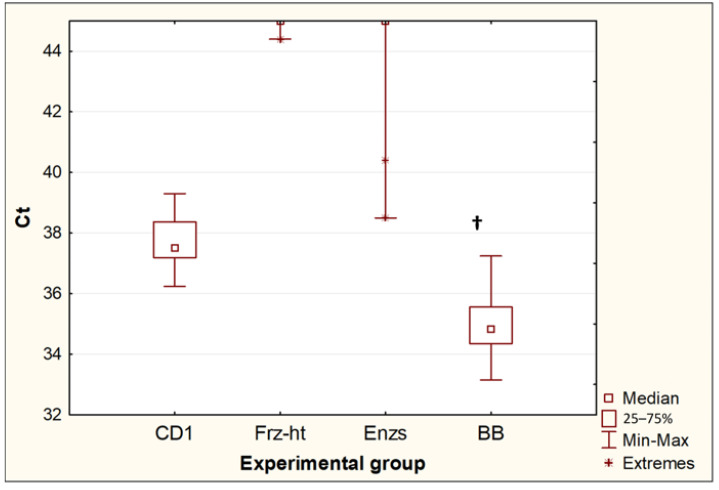
Comparison of threshold amplification cycles (Ct) of DNA after egg disruption by different methods. CD1—incubation in CD1 buffer from the QIAamp PowerFecal Pro kit (*n* = 10); Frz-ht—freeze-heat cycles (*n* = 10); Enzs—treatment with temperature-dependent enzymes from the forensicGEM Sperm kit (*n* = 10); BB—bead beating with beads from the QIAamp PowerFecal Pro kit (*n* = 10); †—*p* < 0.05 vs. CD1.

Of the four methods for destroying *Toxocara* eggs, only bead beating and incubation in CD1 buffer from the QIAamp PowerFecal Pro kit resulted in the release of *Toxocara* DNA in all repeats (10 out of 10 for each experimental group). When comparing these two methods (Figure 3), bead beating resulted in a significant decrease in Ct values (*p* = 0.0005). The difference between the median Ct values was 2.7, which suggests that bead beating increased the yield of DNA from *Toxocara* eggs by approximately 6.4 times. Two other methods, freeze-heat cycles and the incubation of *Toxocara* eggs with temperature-dependent enzymes, only sporadically resulted in DNA release at low concentrations. After the freeze-heat cycles, a positive PCR result was obtained for one sample out of 10 (Ct = 44.4). After the incubation with enzymes from the forensicGEM Sperm kit, only 2 out of 10 samples gave a positive PCR result (Ct = 38.5; 40.4).

### 3.3. Effect of Double Prewash on the PCR Results in the Extraction of T. canis DNA from Native Feces

**Figure 4:**
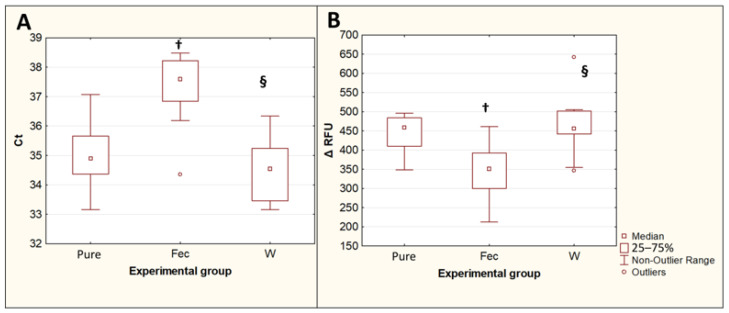
Comparison of PCR results for isolation of *T. canis* DNA from native feces and from feces after double prewashing. (**A**)—threshold amplification cycles (Ct). (**B**)—maximum fluorescence intensity (ΔRFU). Pure—pure suspension of *Toxocara* eggs (*n* = 10); Fec—feces contaminated with *Toxocara* eggs (*n* = 10); W—feces contaminated with *Toxocara* eggs after double preliminary washing (*n* = 10). †—*p* < 0.05 vs. Susp; §—*p* < 0.05 vs. Fec.

The extraction of *T. canis* DNA from contaminated feces using the QIAamp PowerFecal Pro kit for PCR showed an increase in Ct values (*p* = 0.015) and a decrease in the maximum fluorescence intensity ΔRFU (*p* = 0.019) compared with DNA isolated with the same kit from the same number of eggs in the pure *Toxocara* egg suspension. The difference between the median Ct values for the DNA isolated from feces and the DNA isolated from a pure *Toxocara* egg suspension was 2.7, which implies that, when DNA is isolated from feces, without their preliminary purification from inhibitors, the amount of *T. canis* DNA detected by PCR decreases by approximately 6.4 times. The preliminary double washing of the feces contaminated with *T. canis* eggs with a 0.1% solution of Tween-20 in PBS significantly reduced the Ct values (*p* = 0.0013) and increased the ΔRFU (*p* = 0.0082), bringing these values closer to the values obtained by isolating DNA from a pure suspension of *Toxocara* eggs (Figure 4).

### 3.4. Influence of Preconcentration of Helminth Eggs on PCR Results in Isolation of T. canis DNA from Native Feces

**Figure 5:**
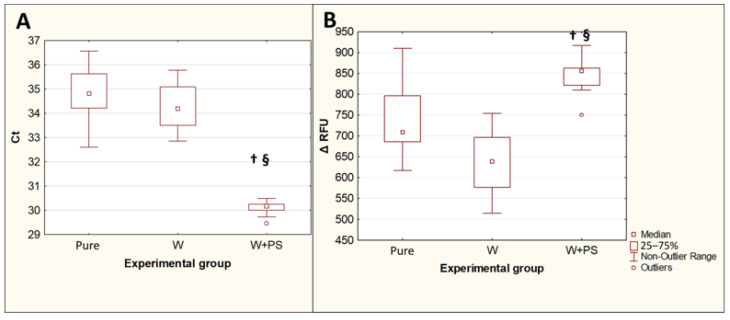
Comparison of the PCR results for isolation of *T. canis* DNA from feces after a double prewash and using a concentrator followed by a single wash. (**A**)—threshold amplification cycles (Ct). (**B**)—maximum fluorescence intensity (ΔRFU). Pure—pure *Toxocara* egg suspension (*n* = 10); W—feces contaminated with *Toxocara* eggs after preliminary double washing (*n* = 10); W + PS—feces contaminated with *Toxocara* eggs after preconcentration followed by single washing (*n* = 10). †—*p* < 0.05 vs. Susp; §—*p* < 0.05 vs. W.

Replacing the first of the two prewashes with the Apacor Parasep mini concentrator significantly reduced the Ct fluorescence threshold cycles (*p* = 0.0016) and increased the maximum fluorescence intensity ΔRFU (*p* = 0.00007) compared to the isolation of *Toxocara* DNA from contaminated and double prewashed feces (Figure 5). The difference between the median Ct values for these two experimental groups was 4, which implies that, when DNA is isolated from feces, after preliminary concentration, the amount of *Toxocara canis* DNA detected by PCR increases by approximately 16 times.

### 3.5. Evaluation of the Minimum Detectable Number of Eggs of Toxocara canis in Native Feces by Real-Time PCR Using the Designed Protocol for the Isolation of STHs DNA from Feces

This experiment used fecal samples contaminated with *T. canis* eggs at concentrations of 1000, 500, 50, 10, and 5 eggs/g, each in 10 repeats.

**Figure 6:**
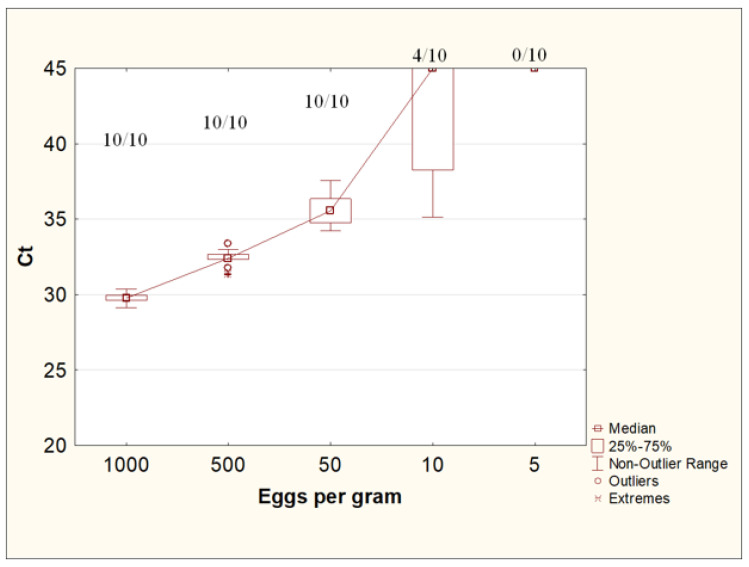
Ct values for DNA isolation from feces contaminated with *Toxocara canis* eggs at various concentrations. The numbers at the top of the graph are the ratio of positive samples according to the results of the PCR study to the total number of samples in the group.

The use of the W + PS protocol with a preliminary concentration of helminth eggs with an Apacor Parasep mini concentrator, washing with 0.1% Tween-20 solution in PBS, and further DNA isolation from feces using the QIAamp PowerFecal Pro kit made it possible to reproducibly detect *Toxocara* DNA in all repeats at concentrations of *Toxocara* eggs of up to 50 eggs per gram of feces. At a concentration of 10 eggs per gram of feces, *Toxocara* DNA was detected in 40% of the samples (4 out of 10). A concentration of five eggs per gram of stool was insufficient to detect *T. canis* DNA by real-time PCR (Figure 6).

## 4. Discussion

The *Toxocara* eggshell has a similar composition and type of structure to eggshells of other STHs that are found in human feces [22,23], which allows for extrapolation of the results of this study to other STH species whose eggs are found in human feces. At the same time, since *T. canis* does not reach the sexually mature stage in the human body and, therefore, cannot produce eggs in the intestine [30], the use of *Toxocara* eggs as a model object completely excluded their presence in fecal samples from humans before the contamination procedure and made it possible to exactly provide the declared concentration of eggs in different fecal samples.

Experiments that compared different methods of destroying *Toxocara* eggs showed that bead beating significantly increased the yield of DNA from the eggs. These data are consistent with the results of previous studies both on helminths of the genus *Toxocara* [31] and on other STHs [32]. Concurrently, the widely used destruction method of exposing helminth eggs to high and low temperatures [33,34,35] did not lead to a significant increase in the number of destroyed eggs observed by microscopy or to a significant release of *T. canis* DNA from the eggs. 

The shells of SHT eggs are largely composed of protein; therefore, proteinases are often used to destroy them [14,36]. For this reason, we decided to test temperature-dependent proteinases from the forensicGEM Sperm kit [37], which are potentially capable of effectively destroying the walls of STH eggs. However, the effectiveness of temperature-dependent enzymes on the destruction of the eggs and the release of helminth DNA has not been shown. In protocols that use enzymatic treatment of helminth eggs, incubation with the enzymes takes from several hours to almost a day [36,38]. This far exceeds the time required for the complete destruction of STH eggs by the bead beating method demonstrated in this study. 

Unexpectedly, there was a discrepancy between the results on the action of the CD1 lysis buffer from the QIAamp PowerFecal Pro kit as obtained by microscopy (the number of undamaged eggs did not change significantly) and by real-time PCR (*T. canis* DNA was detected). The presence of *T. canis* DNA in the samples with a constant number of undamaged eggs can be explained by two reasons. First, in addition to eggs, fragments of helminth tissues could be present in the suspension. In this case, the CD1 buffer could lyse these particles and release their DNA, leaving the eggs intact. The second explanation may be that *Toxocara* eggs, when incubated in CD1 buffer, undergo damage that is not visible under a light microscope but is sufficient to release a significant amount of DNA into the solution. QIAGEN does not disclose the exact composition of its solutions; however, from the documents for the QIAamp PowerFecal Pro kit published on the company’s website [39], it can be concluded that the main lysing component of the CD1 solution is the relatively mild chaotropic agent sodium thiocyanate. According to patent CA3096461A1 from Qiagen Sciences LLC, dedicated to the isolation of nucleic acids and the removal of inhibitors from complex samples, the use of mild lysing chemical agents makes it possible to reduce the loss of isolated nucleic acids but requires additional physical treatment of the sample, for example, by bead beating [40]. However, short-term incubation in a CD1 solution for 5 min, in which the samples were subjected to freeze-heat cycles and after the action of temperature-dependent enzymes, did not lead to a significant yield of DNA. Damage to *Toxocara* eggs can be detected, for example, by immunohistochemistry methods, but we did not use these methods since this was not the main purpose of this study. However, based on the data that were obtained, we can conclude that the bead beating procedure may be sufficient for the destruction of STH eggs, while additional steps for egg destruction, such as temperature or enzyme action, are not necessary.

Despite this result, data on the effect of preliminary washings of feces with 0.1% Tween-20 solution in PBS show that, when using this kit for the isolation of STHs’ DNA from feces, it is desirable to use additional steps which are aimed at removing PCR inhibitors. Although, in the experiment on the effect of preliminary washings of feces in all experimental groups, the number of *Toxocara* eggs was approximately the same (approximately 200 eggs per sample), the amount of *T. canis* DNA that was detected when it was isolated from feces was several times lower than when it was isolated from a pure egg suspension. A significant decrease in ΔRFU was also noted when the DNA was isolated from contaminated feces compared with a pure *Toxocara* egg suspension. The ΔRFU depends on many parameters of the reaction mixture, including the fluorescent label used, the ionic strength of the solution, the cycler parameters, etc. Having said that, a decrease in the ΔRFU when using the same reagents within one setting on the device may indicate the presence of PCR inhibitors in the sample [41,42]. At the same time, preliminary washings of fecal samples with a simple solution of a nonionic surfactant in a buffer can significantly reduce PCR inhibition, which is expressed by a decrease in the Ct value and an increase in the ΔRFU to the levels that are achieved when *Toxocara* eggs are isolated from a pure suspension of eggs. Thus, the prewashing of fecal samples before the isolation of parasitic DNA is an important step that can significantly improve the PCR results.

Fluorescence quenching resulting in a decrease in ΔRFU is an important indicator of PCR inhibition because it does not always correlate with changes in the Ct value. The reasons for the Ct value changing can be both PCR inhibition and the loss of DNA during the extraction process. In contrast, the change in ΔRFU depends on the amount of inhibitors, rather than the amount of target DNA in the sample. Mechanisms for reducing ΔRFU include direct interference with the fluorophore and inhibition of the 5′–3′-exonuclease activity of the polymerase. As a result, the polymerase displaces the probe from the duplex complex without hydrolyzing it [43]. Fluorescence inhibition is an overlooked phenomenon that needs to be considered to allow for the development of optimal qPCR assays [44].

The concentration of STH eggs before DNA isolation can significantly increase the sensitivity of molecular diagnostic methods for helminth infections in low-incidence regions, where patients with a low helminth invasion range often have relatively few parasite eggs in their feces. The use of commercially available parasite concentrators for feces is a fairly common practice in parasitological studies that use microscopy methods [45,46]. We have found a small number of works where protocols for isolating helminth DNA used flotation–sedimentation for concentrating parasite eggs from feces [47,48,49] and sewage sludge [50]. Nonetheless, in most of these works, the eggs were washed off coverslips after flotation for further DNA isolation, or from sieves, in the case of sewage sludge. This increases the risk of losing part of the eggs of parasites, and significantly complicates the procedure for DNA extraction, which makes these methods of isolation unsuitable for use in the routine laboratory diagnosis of helminthiases.

In addition, these works did not evaluate the effectiveness of the removal of PCR inhibitors by the flotation methods. Studies on the use of commercially available parasite egg concentrators for the isolation of STH DNA from feces are not found in the literature. 

Theoretically, in addition to physical methods, it is possible to concentrate STH eggs from feces using immunosorbent methods. The literature describes protocols for isolating the DNA of protozoan parasites with concentration using the method of immunomagnetic separation [51]. However, this method is quite laborious and much more expensive than the parasite concentrator, and, before its use, a step of preliminary isolation of parasite cysts from feces is still necessary, for example, by flotation. In addition, the existing commercially available kits are designed for the immunomagnetic separation of protozoan parasites (*Cryptosporidium* spp., *Giardia lamblia*, etc.). The isolation of STH eggs by this method is likely to be less efficient due to the much larger size of helminth eggs compared to parasitic protozoan cysts. 

We chose the PCR reaction parameters as the main indicators of the DNA extraction effectivity and did not use methods such as measuring the total DNA amount using NanoDrop. We preferred PCR because, compared with NanoDrop, PCR provides a measure of only the target *T. canis* DNA. NanoDrop, in contrast, measures the total amount of DNA, consisting mainly of bacterial DNA after being isolated from feces. Moreover, unlike NanoDrop, it is possible to compare the amount of inhibitors after different DNA extraction methods using a PCR.

In the experiment on the effect of the concentration of helminth eggs, in the group with the replacement of the first wash with Apacor Parasep Mini concentrators, due to the ability to use a larger volume of feces for DNA extraction, the number of *T. canis* eggs was approximately 500 eggs per sample, which was 2.5 times higher than the number of eggs per sample in the two-wash group (approximately 200 eggs per sample). Simultaneously, the amount of detectable *T. canis* DNA which was obtained by adding the concentration step to the DNA isolation protocol increased by an average of 16 times. This suggests that the use of concentrators not only allows the number of STHs eggs to increase in the test sample but also, additionally, reduces the amount of PCR inhibitors in the sample. At the same time, one washing of the precipitate which was obtained during the concentration with a Tween-20 solution is sufficient to remove the formalin from the precipitate, which is part of the solution used in the concentrator and is a powerful PCR inhibitor [52]. In addition, the use of a commercial concentrator does not take much time and they are easy to use, since they do not require the manipulation of additional flotation solutions, filters, or sieves.

According to the present study, the use of a modified protocol for the QIAamp PowerFecal Pro excretion kit, with a preliminary concentration of the helminth eggs with an Apacor Parasep mini concentrator and a single wash of 0.1% solution of Tween-20 in PBS, made it possible to detect *T. canis* DNA in 100% of the samples at concentrations up to 50 eggs per gram of feces and in 40% of the samples at a concentration of 10 eggs per gram of feces. For *T. canis*, the mean values for eggs in dog feces range from 400 to 1900 eggs per gram, depending on the age of the dog and the study region [53,54]. For STHs parasitizing the human intestine, according to WHO recommendations, the boundary values for a low intensity of helminthic infection range from 1000 (for *Trichuris trichiura* (Linnaeus, 1771) to 4000 (for *Ascaris lumbricoides* Linnaeus, 1758) eggs per gram of feces [55]. *Toxocara canis* was a model object in our study, and the efficiency of DNA extraction for other STH species may differ. Nevertheless, the obtained results, together with the data presented in the literature, suggest that our proposed protocol has sufficient extraction efficiency to detect human intestinal STH DNA in most cases of mild invasion.

We chose the following protocol for the isolation of STHs’ DNA from feces as the optimal protocol:

1. Unscrew the lid from the sample collection unit of the Apacor Mini Parasep Concentrator (do not discard the lid!), and add approximately 500 mg of fecal sample (1 spoonful) into the compartment using the spoon at the end of the filter.
2. Follow the “Emulsification” and “Centrifugation” sections of the instructions for the Apacor Mini Parasep concentrator kit.
3. Gently open the device and discard the top chamber of the concentrator along with the filter.
4. Discard the supernatant, add 1.5 mL of a 0.1% solution of Tween-20 in PBS to the sediment, and then close the tube with the lid from the sample collection compartment and resuspend the sediment.
5. Centrifuge the tubes for 5 min at 5000× *g* and discard the supernatant.
6. Add 800 µL of CD1 buffer from the QIAamp PowerFecal Pro kit to the sediment and resuspend the sediment on a vortex.
7. Transfer the resulting suspension to a clean PowerBead Pro Tube from the QIAamp PowerFecal Pro kit.
8. Homogenize the samples on the bead beating homogenizer at maximum speed for 30 min.
9. Follow the instructions for the QIAamp PowerFecal Pro kit starting at step 3.

## 5. Conclusions

Thus, in this study, we have shown that bead beating at 50 Hz for 30 min is enough for the destruction of STHs’ eggs. In contrast, such procedures as four freeze-heat cycles (−70–+95 °C) or treatment with temperature-dependent enzymes from the forensicGEM Sperm kit had no significant effect on the *T. canis* DNA yield nor on the egg integrity. Therefore, using protocols for extracting DNA from STH eggs that do not include a bead beating step is not optimal. Steps such as the preconcentration of STH eggs from feces using Apacor Parasep mini concentrators, as well as washing the feces concentrate with a 0.1% Tween-20 nonionic surfactant solution significantly increased the amount of *T. canis* DNA that was extracted from the samples and decreased the amount of PCR inhibitors in the extracted DNA. In summary, we propose a protocol for parasitic helminth DNA extraction from feces, which can be suitable for use in qPCR tests in particular.

## Figures and Tables

**Table 1:** Sample preparation protocols for evaluating the effects of various destructive methods on the integrity of eggs and the efficiency of *Toxocara canis* DNA extraction.

Sample *	Protocols for Assessing Egg Integrity	Protocols for Evaluating the Efficiency of DNA Extraction
Susp	Sample was centrifuged with 100 µL of *Toxocara* egg suspension (750 eggs/mL) at 10,000× *g*, 5 min; Without discarding the supernatant, resuspend the sediment.	The study was not carried out
CD1	To 100 µL of Toxocara egg suspension (750 eggs/mL) 400 µL of CD1 buffer from the QIAamp PowerFecal Pro kit was added; Incubated in a thermoshaker for 30 min, at a speed of 300 rpm at 25 °C; The sample was centrifuged at 10,000× g for 5 min, 400 μL of the supernatant was taken, after which the sediment was resuspended in the remaining liquid.	Added to 10 1.5 mL tubes, 134 µL of *Toxocara* egg suspension (1500 eggs/mL), added 800 µL of CD1 buffer from the QIAamp PowerFecal Pro kit; The tubes were incubated in a thermoshaker for 30 min at a speed of 300 rpm at 25 °C; Further DNA extraction was carried out according to the instructions for the QIAamp PowerFecal Pro kit [24], starting from step 4 (excluding the homogenization stage), the entire content was transferred into 2 mL tubes
Frz-ht	1. The sample was incubated with 100 μL of *Toxocara* egg suspension (750 eggs/mL) in a freezer at −70 °C for 30 min; 2. The sample was incubated in a thermoshaker at a speed of 300 rpm at 95 °C for 10 min; Repeat steps 1 and 2 four times; 3. The sample was centrifuged at 10,000× *g* for 5 min, the sediment was resuspended without discarding the supernatant.	1. Added to 10 1.5 mL test tubes, 134 μL of *Toxocara* egg suspension (1500 eggs/mL); 2. Samples were incubated in a freezer at −70 °C for 30 min; 3. Samples were incubated in a thermoshaker at a speed of 300 rpm at 95 °C for 10 min; Repeat steps 2 and 3 four times; 4. Added 800 µL of the QIAamp PowerFecal Pro kit to the samples, incubated for 5 min at room temperature; 5. Further DNA extraction was carried out according to the instructions for the QIAamp PowerFecal Pro kit [24], starting from step 4 (excluding the homogenization stage), all of the contents were transferred into 2 mL tubes.
Enzs	A sample was centrifuged with 100 µL of a suspension of *Toxocara* eggs (750 eggs/mL) at 10,000× *g*, 5 min, 90 µL of the supernatant was taken, after which the sediment was resuspended in the remaining liquid; Added reagents from the forensicGEM Sperm kit: 10 µL buffer, 10 µL Acrosolv, 2 µL forensicGEM, 68 µL deionized water; Samples were incubated in a thermoshaker at a speed of 300 rpm for 30 min at 52°, then 30 min at 75°, then 10 min at 95°	Added to 10 1.5 mL tubes 134 μL of *Toxocara* egg suspension (1500 eggs/mL); The tube was centrifuged at 10,000× *g* for 5 min, 124 µL of the supernatant was taken, after which the sediment was resuspended; Added reagents from the forensicGEM Sperm kit: 10 µL buffer, 10 µL Acrosolv, 2 µL forensicGEM, 68 µL deionized water; Samples were incubated in a thermoshaker at 300 rpm for 30 min at 52°, then 30 min at 75°, then 10 min at 95°; 800 µL of CD1 buffer from the QIAamp PowerFecal Pro kit was added to the samples and incubated for 5 min at room temperature; Further DNA extraction was carried out according to the instructions for the QIAamp PowerFecal Pro kit [24], starting from step 4 (excluding the homogenization stage), all contents were transferred into 2 mL tubes.
BB	Transferred to the beating tube from the QIAamp PowerFecal Pro kit 100 µL of *Toxocara* egg suspension (750 eggs/mL) and 800 µL of saline was added; The sample was homogenized on a QIAamp TissueLyser homogenizer at 50 Hz for 30 min; Immediately after homogenization, 600 µL of the upper part of the homogenate was taken, without capturing the beads, and transferred to a clean 1.5 mL tube; The tube was centrifuged at 10,000× *g* for 5 min, 500 µL of the supernatant was taken, after which the sediment was resuspended; Added to the same beating tube 600 µL of saline, vortexed for 5 s at maximum speed, after which 600 µL of liquid was taken into the tube with the homogenate sediment; The tube was centrifuged at 10,000× *g* for 5 min, 600 µL of the supernatant was taken, after which the sediment was resuspended in the remaining liquid; Repeat steps 5 and 6 four times	Added to 10 PowerBead Pro Tubes from the QIAamp PowerFecal Pro kit 134 µL of *Toxocara* egg suspension (1500 eggs/mL); 800 µL of CD1 buffer from the QIAamp PowerFecal Pro kit was added to the samples, mixed on a vortex; The samples were homogenized on a QIAamp TissueLyser homogenizer at 50 Hz for 30 min; Further DNA extraction was carried out according to the instructions for the QIAamp PowerFecal Pro kit [24], starting from step 3 (centrifugation step after homogenization).

* The names of samples. Susp—pure suspension (control); CD1—incubation in CD1 buffer of the QIAamp Power-Fecal Pro kit; Frz-ht—four cycles of freezing-heating; Enzs—temperature-dependent enzymes from the forensicGEM Sperm kit; BB—bead beating with QIAamp PowerFecal Pro ceramic beads.

**Table 2:** Primers and probe for *T. canis* detection.

Primer/Probe	Sequence (5′–3′)	Length
Fwd	TTTGCACGTATGCGTGAGCC	20
Rev	GCCTTTCTAACTTGCCCAGC	20
Probe	FAM-CGTCACCCATTTCCCCTCAACAC-BHQ1	23

**Table 3:** DNA extraction protocols for the experiment on the effect of prewashes on PCR results.

Group *	Protocol
Pure (*n* = 10)	Ten PowerBead Pro Tubes from the QIAamp PowerFecal Pro kit had 134 µL of *Toxocara* egg suspension added; 800 µL of CD1 buffer from the QIAamp PowerFecal Pro kit was added to the samples, and mixed on a vortex; The samples were homogenized on a QIAamp TissueLyser homogenizer at 50 Hz for 30 min; Further stages of DNA extraction were carried out according to the instructions for the QIAamp PowerFecal Pro kit [24], starting from step 3.
Fec (*n* = 10)	Ten PowerBead Pro Tubes from the QIAamp PowerFecal Pro kit were filled with 200 mg of feces contaminated with *Toxocara* eggs at a concentration of 1000 eggs/g; 800 µL of CD1 buffer from the QIAamp PowerFecal Pro kit was added to the samples, and mixed on a vortex; The samples were homogenized on a QIAamp TissueLyser homogenizer at 50 Hz for 30 min; Further stages of DNA extraction were carried out according to the instructions for the QIAamp PowerFecal Pro kit [24], starting from step 3 (the stage of sample centrifugation after homogenization).
W (*n* = 10)	1. Ten 2 mL test tubes were filled with 200 mg of feces contaminated with *Toxocara* eggs at a concentration of 1000 eggs/g; 0.5 mL of a 0.1% solution of Tween-20 in PBS was added to the tubes, after which the tubes were vortexed until the feces were completely dissolved; The tubes were centrifuged for 5 min at 5000× *g*, after which the supernatant was merged; Steps 2 and 3 were repeated 2 times 2. The sediment was resuspended in 800 µL of CD1 buffer from the QIAamp PowerFecal Pro kit, vortexed, and then the contents of the tubes were transferred to clean PowerBead Pro Tubes from the QIAamp PowerFecal Pro kit; 3. The samples were homogenized on a QIAamp TissueLyser homogenizer at 50 Hz for 30 min; 4. Further stages of DNA extraction were carried out according to the instructions for the QIAamp PowerFecal Pro kit [24], starting from step 3 (centrifugation of the samples after homogenization).

* The names of groups. Pure—pure suspension of *Toxocara* eggs; Fec—fecal samples contaminated with *Toxocara* eggs without prewash; W—fecal samples contaminated with *Toxocara* eggs at a concentration of 1000 eggs/g, preliminarily washed twice with 0.1% Tween-20.

## Data Availability

All raw data is available at https://osf.io/2ruyt/files/osfstorage (accessed on 1 May 2023).

## References

[1] de Silva N.R., Brooker S., Hotez P.J., Montresor A., Engels D., Savioli L. (2003). Soil-Transmitted Helminth Infections: Updating the Global Picture. Trends Parasitol..

[2] Pullan R.L., Smith J.L., Jasrasaria R., Brooker S.J. (2014). Global Numbers of Infection and Disease Burden of Soil Transmitted Helminth Infections in 2010. Parasit. Vectors.

[3] Nokes C., Grantham-McGregor S.M., Sawyer A.W., Cooper E.S., Robinson B.A., Bundy D.A. (1992). Moderate to Heavy Infections of *Trichuris trichiura* Affect Cognitive Function in Jamaican School Children. Parasitology.

[4] Savioli L., Bundy D., Tomkins A. (1992). Intestinal Parasitic Infections: A Soluble Public Health Problem. Trans. R. Soc. Trop. Med. Hyg..

[5] Cools P., Vlaminck J., Albonico M., Ame S., Ayana M., José Antonio B.P., Cringoli G., Dana D., Keiser J., Maurelli M.P. (2019). Diagnostic Performance of a Single and Duplicate Kato-Katz, Mini-FLOTAC, FECPAKG2 and QPCR for the Detection and Quantification of Soil-Transmitted Helminths in Three Endemic Countries. PLoS Negl. Trop. Dis..

[6] Endris M., Tekeste Z., Lemma W., Kassu A. (2013). Comparison of the Kato-Katz, Wet Mount, and Formol-Ether Concentration Diagnostic Techniques for Intestinal Helminth Infections in Ethiopia. ISRN Parasitol..

[7] Mengist H.M., Demeke G., Zewdie O., Belew A. (2018). Diagnostic Performance of Direct Wet Mount Microscopy in Detecting Intestinal Helminths among Pregnant Women Attending Ante-Natal Care (ANC) in East Wollega, Oromia, Ethiopia. BMC Res. Notes.

[8] Calderaro A., Montecchini S., Rossi S., Gorrini C., De Conto F., Medici M.C., Chezzi C., Arcangeletti M.C. (2014). Intestinal Parasitoses in a Tertiary-Care Hospital Located in a Non-Endemic Setting during 2006–2010. BMC Infect. Dis..

[9] Benjamin-Chung J., Pilotte N., Ercumen A., Grant J.R., Maasch J.R.M.A., Gonzalez A.M., Ester A.C., Arnold B.F., Rahman M., Haque R. (2020). Comparison of Multi-Parallel QPCR and Double-Slide Kato-Katz for Detection of Soil-Transmitted Helminth Infection among Children in Rural Bangladesh. PLoS Negl. Trop. Dis..

[10] Liu C., Lu L., Zhang L., Bai Y., Medina A., Rozelle S., Smith D.S., Zhou C., Zang W. (2017). More Poop, More Precision: Improving Epidemiologic Surveillance of Soil-Transmitted Helminths with Multiple Fecal Sampling Using the Kato–Katz Technique. Am. J. Trop. Med. Hyg..

[11] Bärenbold O., Raso G., Coulibaly J.T., N’Goran E.K., Utzinger J., Vounatsou P. (2017). Estimating Sensitivity of the Kato-Katz Technique for the Diagnosis of *Schistosoma mansoni* and Hookworm in Relation to Infection Intensity. PLoS Negl. Trop. Dis..

[12] O’Connell E.M., Nutman T.B. (2016). Molecular Diagnostics for Soil-Transmitted Helminths. Am. J. Trop. Med. Hyg..

[13] Knopp S., Salim N., Schindler T., Karagiannis Voules D.A., Rothen J., Lweno O., Mohammed A.S., Singo R., Benninghoff M., Nsojo A.A. (2014). Diagnostic Accuracy of Kato–Katz, FLOTAC, Baermann, and PCR Methods for the Detection of Light-Intensity Hookworm and *Strongyloides stercoralis* Infections in Tanzania. Am. J. Trop. Med. Hyg..

[14] Diawara A., Drake L.J., Suswillo R.R., Kihara J., Bundy D.A.P., Scott M.E., Halpenny C., Stothard J.R., Prichard R.K. (2009). Assays to Detect β-Tubulin Codon 200 Polymorphism in *Trichuris trichiura* and *Ascaris lumbricoides*. PLoS Negl. Trop. Dis..

[15] Al-Soud W.A., Ouis I.-S., Li D.-Q., Ljungh S., Wadström T. (2005). Characterization of the PCR Inhibitory Effect of Bile to Optimize Real-Time PCR Detection of *Helicobacter* Species. FEMS Immunol. Med. Microbiol..

[16] Repetto S.A., Alba Soto C.D., Cazorla S.I., Tayeldin M.L., Cuello S., Lasala M.B., Tekiel V.S., González Cappa S.M. (2013). An Improved DNA Isolation Technique for PCR Detection of *Strongyloides stercoralis* in Stool Samples. Acta Trop..

[17] Sharifdini M., Mirhendi H., Ashrafi K., Hosseini M., Mohebali M., Khodadadi H., Kia E.B. (2015). Comparison of Nested Polymerase Chain Reaction and Real-Time Polymerase Chain Reaction with Parasitological Methods for Detection of *Strongyloides stercoralis* in Human Fecal Samples. Am. J. Trop. Med. Hyg..

[18] Leles D., Araújo A., Vicente A.C.P., Iñiguez A.M. (2009). Molecular Diagnosis of Ascariasis from Human Feces and Description of a New *Ascaris* Sp. Genotype in Brazil. Vet. Parasitol..

[19] Sultana Y., Jeoffreys N., Watts M.R., Gilbert G.L., Lee R. (2013). Real-Time Polymerase Chain Reaction for Detection of *Strongyloides stercoralis* in Stool. Am. J. Trop. Med. Hyg..

[20] Verweij J.J., Stensvold C.R. (2014). Molecular Testing for Clinical Diagnosis and Epidemiological Investigations of Intestinal Parasitic Infections. Clin. Microbiol. Rev..

[21] Zou Y., Zheng W.-B., He J.-J., Elsheikha H.M., Zhu X.-Q., Lu Y.-X. (2020). *Toxocara canis* Differentially Affects Hepatic MicroRNA Expression in Beagle Dogs at Different Stages of Infection. Front. Vet. Sci..

[22] Mkandawire T.T., Grencis R.K., Berriman M., Duque-Correa M.A. (2022). Hatching of Parasitic Nematode Eggs: A Crucial Step Determining Infection. Trends Parasitol..

[23] Wharton D. (1980). Nematode Egg-Shells. Parasitology.

[24] QIAamp PowerFecal Pro DNA Kit Handbook. https://www.qiagen.com/us/Resources/ResourceDetail?id=8896817a-253f-4952-b845-0aab796813ce&lang=en.

[25] NCBI Primer-Blast Primer Designing Tool. https://www.ncbi.nlm.nih.gov/tools/primer-blast/.

[26] Home—Nucleotide—NCBI. https://www.ncbi.nlm.nih.gov/nuccore.

[27] van Pelt-Verkuil E., van Belkum A., Hays J.P. (2008). Principles and Technical Aspects of PCR Amplification.

[28] Basu C. (2015). PCR Primer Design.

[29] The Mfold Web Server. http://www.unafold.org/mfold/applications/dna-folding-form.php.

[30] Garcia L.S. (2007). Diagnostic Medical Parasitology.

[31] Mikaeili F., Kia E.B., Sharbatkhori M., Sharifdini M., Jalalizand N., Heidari Z., Zarei Z., Stensvold C.R., Mirhendi H. (2013). Comparison of Six Simple Methods for Extracting Ribosomal and Mitochondrial DNA from *Toxocara* and *Toxascaris* Nematodes. Exp. Parasitol..

[32] Andersen L.O., Röser D., Nejsum P., Nielsen H.V., Stensvold C.R. (2013). Is Supplementary Bead Beating for DNA Extraction from Nematode Eggs by Use of the NucliSENS EasyMag Protocol Necessary?. J. Clin. Microbiol..

[33] George S., Geldhof P., Albonico M., Ame S.M., Bethony J.M., Engels D., Mekonnen Z., Montresor A., Hem S., Tchuem-Tchuenté L.-A. (2017). The Molecular Speciation of Soil-Transmitted Helminth Eggs Collected from School Children across Six Endemic Countries. Trans. R. Soc. Trop. Med. Hyg..

[34] Cunningham L.J., Odoom J., Pratt D., Boatemaa L., Asante-Ntim N., Attiku K., Banahene B., Osei-Atweneboana M., Verweij J.J., Molyneux D. (2018). Expanding Molecular Diagnostics of Helminthiasis: Piloting Use of the GPLN Platform for Surveillance of Soil Transmitted Helminthiasis and Schistosomiasis in Ghana. PLoS Negl. Trop. Dis..

[35] Ayana M., Cools P., Mekonnen Z., Biruksew A., Dana D., Rashwan N., Prichard R., Vlaminck J., Verweij J.J., Levecke B. (2019). Comparison of Four DNA Extraction and Three Preservation Protocols for the Molecular Detection and Quantification of Soil-Transmitted Helminths in Stool. PLoS Negl. Trop. Dis..

[36] Khademvatan S., Abdizadeh R., Tavalla M. (2014). Molecular Characterization of *Toxocara* Spp. from Soil of Public Areas in Ahvaz Southwestern Iran. Acta Trop..

[37] ForensicGEM Sperm Product Overview. https://microgembio.com/product/forensicgem-sperm-dna-extraction-kit/#s-overview.

[38] Collender P.A., Kirby A.E., Addiss D.G., Freeman M.C., Remais J. (2015). V Methods for Quantification of Soil-Transmitted Helminths in Environmental Media: Current Techniques and Recent Advances. Trends Parasitol..

[39] QIAamp PowerFecal Pro DNA Kits For the Isolation of Microbial DNA from Stool and Gut Samples. https://www.qiagen.com/us/products/discovery-and-translational-research/dna-rna-purification/dna-purification/genomic-dna/qiaamp-powerfecal-pro-dna-kit/.

[40] Callahan H., Nieciecki V., Deforce E., Adams E.W. (2019). Nucleic Acid Isolation and Inhibitor Removal from Complex Samples. U.S. Patent.

[41] Maddocks S., Jenkins R. (2017). Quantitative PCR. Understanding PCR.

[42] Do the Levels of Relative Fluorescence Units (RFUs) for a QPCR Reaction Have an Impact on My Data?|Bio-Rad. https://www.bio-rad.com/ru-ru/faq/Do-levels-of-RFUs-fo_1384541547/normalization-of-real-time-pcr-fluorescence-data-with-rox-passive-reference-dye.

[43] Sidstedt M., Rådström P., Hedman J. (2020). PCR Inhibition in QPCR, DPCR and MPS—Mechanisms and Solutions. Anal. Bioanal. Chem..

[44] Sidstedt M., Jansson L., Nilsson E., Noppa L., Forsman M., Rådström P., Hedman J. (2015). Humic Substances Cause Fluorescence Inhibition in Real-Time Polymerase Chain Reaction. Anal. Biochem..

[45] Mewara A., Khurana S., Gupta S., Munda V.S., Singh S., Sehgal R. (2019). Diagnostic Performance of Mini Parasep^®^ Solvent-Free Foecal Parasite Concentrator for the Diagnosis of Intestinal Parasitic Infections. Indian J. Med. Microbiol..

[46] Adugna S., Kebede T., Mekonnen Z., Degarege A., Liang S., Erko B. (2017). Diagnostic Performance of Mini Parasep® Solvent-Free Faecal Parasite Concentrator Relative to Kato-Katz and McMaster for the Diagnosis of Intestinal Parasitic Infections. Trans. R. Soc. Trop. Med. Hyg..

[47] Phadungsil W., Pumpa S., Sirisabhabhorn K., Geadkaew-Krenc A., Grams R., Mungthin M., Ruang-Areerate T., Adisakwattana P., Labbunruang N., Martviset P. (2021). Efficiency of the Stool-PCR Test Targeting NADH Dehydrogenase (Nad) Subunits for Detection of *Opisthorchis viverrini* Eggs. J. Trop. Med..

[48] Wang N., Wang Y., Ye Q., Yang Y., Wan J., Guo C., Zhan J., Gu X., Lai W., Xie Y. (2018). Development of a Direct PCR Assay to Detect *Taenia multiceps* Eggs Isolated from Dog Feces. Vet. Parasitol..

[49] Zarlenga D.S., Barry Chute M., Gasbarre L.C., Boyd P.C. (2001). A Multiplex PCR Assay for Differentiating Economically Important Gastrointestinal Nematodes of Cattle. Vet. Parasitol..

[50] Amoah I.D., Singh G., Troell K., Reddy P., Stenström T.A., Bux F. (2020). Comparative Assessment of DNA Extraction Procedures for *Ascaris* Spp. Eggs. J. Helminthol..

[51] Coklin T., Farber J.M., Parrington L.J., Kingombe C.I.B., Ross W.H., Dixon B.R. (2011). Immunomagnetic Separation Significantly Improves the Sensitivity of Polymerase Chain Reaction in Detecting *Giavdia duodenalis* and *Cryptosporidium* spp. in Dairy Cattle. J. Vet. Diagnostic Investig..

[52] Vitošević K., Todorović M., Varljen T., Slović Ž., Matić S., Todorović D. (2018). Effect of Formalin Fixation on Pcr Amplification of DNA Isolated from Healthy Autopsy Tissues. Acta Histochem..

[53] Sowemimo O.A. (2007). Prevalence and Intensity of *Toxocara canis* (Werner, 1782) in Dogs and Its Potential Public Health Significance in Ile-Ife, Nigeria. J. Helminthol..

[54] Bizhga B., Boçar A., Shehdula D., Shabani E., Rugji J., Roko X., Kosova R. (2014). *Toxocara canis* in Stray Dogs of Tirana and Related Public Health Risks. Paripex—Indian J. Res..

[55] Montresor A., Crompton D.W.T., Hall A., Bundy D.A.P., Savioli L. (1998). Guidelines for the Evaluation of Soil-Transmitted Helminthiasis and Schistosomiasis at Community Level: A Guide for Managers of Control Programmes.

